# Effects of Fish Protein Hydrolysate on the Nutritional, Rheological, Sensorial, and Textural Characteristics of Bread

**DOI:** 10.3390/foods13050698

**Published:** 2024-02-25

**Authors:** Oana Bianca Oprea, Sigurd Sannan, Ignat Tolstorebrov, Ingrid Camilla Claussen, Liviu Gaceu

**Affiliations:** 1 Faculty of Food and Tourism, Transilvania University of Brasov, Castelului 148, 500014 Brașov, Romania; oprea.oana.bianca@unitbv.ro; 2 SINTEF Energi AS, Postboks 4761 Torgarden, 7465 Trondheim, Norway; 3 NTNU, Institutt for Energi- og Prosessteknikk, Postboks 8900 Torgarden, 7491 Trondheim, Norway; 4 SINTEF Ocean AS, Postboks 4760 Torgarden, 7465 Trondheim, Norway; 5 CSCBAS&CE-MONT Centre/INCE-Romanian Academy, Casa Academiei Române, Calea 13 Septembrie No. 13, 050711 Bucharest, Romania; 6 Academy of Romanian Scientists, Ilfov Street, No. 3, 050044 Bucharest, Romania

**Keywords:** fish protein hydrolysate, bakery industry, Mixolab, food water footprint

## Abstract

The potential enhancement of the protein content in bakery products is studied by adding fish protein hydrolysate (FPH) flour in varying proportions (1.5%, 3%, 4.5%, and 6%) within the production recipe. The mixtures of wheat flour and FPH obtained were comprehensively analysed using Mixolab equipment, evaluating the nutritional, rheological, and enzymatical aspects. The results underscore the substantial potential of FPH as a high-quality protein source evidenced by its polyphenol content and antioxidant value. Moreover, the utilisation of hydrolysed proteins from fish emerges as a viable strategy for reducing the water footprint in food production. Thus, FPH flour showed a protein content of 80.21%, a polyphenol content of 1452 mg GAE/100 g, and an antioxidant activity of 294 mg TE/100 g. While the bread samples made from wheat flour mixed with FPH exhibited a satisfactory rheological behaviour, the presence of an aftertaste and the pronounced fish aroma impacted consumer acceptance. Notably, only the bread sample with 1.5% added FPH met the organoleptic preferences of the consumers, receiving a commendable total acceptability score of 6.2. Additionally, this sample demonstrated favourable results in texture analysis and exhibited an extended shelf life compared to that of the control sample.

## 1. Introduction

Globally, approximately one-third of the food produced for human consumption goes to waste each year [1,2]. While more than 40% of the food losses in industrialised countries occur at retail and consumer levels [3], more than half of the losses take place up-stream in the food supply chain. Food waste has significant climate and environmental impacts due to increased emissions in the supply chain, waste disposal, and unnecessary use of energy, water, and land. This also has a negative impact economically in terms of the associated costs [4], for food security, and for securing political and economic/societal development in the world. Furthermore, the world population growth, projected to reach 9.6 billion by 2050 [5,6], intensifies the demand for food, implying that food sustainability is a major global concern.

According to the Food and Agriculture Organization (FAO), the global fish production was estimated to be about 179 million tonnes in 2018, of which 82 million tonnes came from aquaculture production [7]. Out of these total production, around 156 million tonnes were allocated for human consumption, while 22 million tonnes served various other purposes, predominantly fishmeal and fish oil production [7]. Notably, an estimated 25–35% of fishmeal and fish oil is produced from the by-products of fish processing [7,8]. Fishmeal and fish oil can also be produced using whole fish, primarily from small pelagic species [9,10].

Nonetheless, significant amounts of fish processing by-products are discarded annually [11,12]. Every year, more than 91 million tonnes of fish are harvested, with 29.5% of this yield being transformed into fishmeal [9,13]. Remarkably, over 50% of the remaining fish tissue are classified as waste and go unused for human consumption [14,15]. These inedible components, encompassing bones, skin/scales, swim bladder, fins, intestines, blood, roe, liver, etc., are often dismissed as waste. However, these tissues are also valuable sources of nutrients, including proteins, lipids, bioactive peptide enzymes, pigments, flavours, vitamins, and minerals. To mitigate the risk of environmental degradation, it is imperative to recycle these valuable wastes into commercially viable items [2,16,17]. For finfish, typical by-products include trimmings, skins, heads, frames (bones with attached flesh), viscera (guts), and blood. Stevens et al. [18] provided a breakdown of the by-product fractions as a percentage of the total wet weight of Atlantic salmon: viscera (12.5%), heads (10%), frames (10%), skins (3.5%), blood (2%), trimming (2%), and belly flap (1.5%) [19].

Numerous studies on the valorisation of fish processing by-products have resulted in FPH with exceptional functional properties. FPH is a valuable component within the family of dried minced fish products, distinguishing itself from fish protein concentrates and isolates due to its enhanced digestibility. The hydrolysis process breaks down proteins into small peptides and amino acids, offering superior functional properties and high nutritional value, including bioactivities like antihypertensive, antithrombotic, immunomodulatory, and antioxidative effects [20].

FPH production involves fractioning raw materials into peptides using either chemical or enzymatic methods. The process offers advantages such as continuous processing and high yields; yet, it comes with challenges including bitterness, smell, lipid oxidation, and stability issues. The schematic of the production process includes thawing, mincing, homogenisation, hydrolysis initiation, termination, solid-liquid separation, pasteurisation, concentration, drying, and packaging. Although spray drying is the predominant industrial drying method [21], freeze drying is also a viable option that results in low moisture rates and good protein quality [22].

Chemical hydrolysis, despite its historical use for simplicity and cost-effectiveness, has limitations in terms of control and consistency. Enzymatic hydrolysis is a more advanced method that offers better control over process parameters, resulting in improved nutritional qualities and functionality. Selecting suitable enzymes, managing water content, and controlling temperature are crucial aspects. Active endogenous enzymes and commercial enzymes (animal, plant, or microbial origin) are employed based on efficacy and economic considerations [23]. The functional properties of FPH, including solubility, water-binding capacity, foam, and emulsion stability, are vital for applications in sports nutrition and health foods. However, producers face challenges such as bitter taste, fishy smell, and variable sensory properties [24]. The degree of hydrolysis (DH) is a key parameter affecting functional properties, with studies indicating a correlation between DH, peptide size, and taste [22].

The composition of FPH varies depending on the raw material and processing techniques, with protein content ranging from 60 to 90%, fat below 10%, moisture below 10%, and ash content between 0.45% and 27% [20]. Energy consumption in FPH manufacturing primarily occurs during hydrolysis, concentration, and drying, with the drying step being the main energy consumer. Industrial spray dryers, commonly used for drying applications, have an average energy consumption of 4880–11,500 kJ/kg of evaporated water.

The aim of this article is to evaluate the nutritional, rheological, organoleptic, and textural potential of using FPH flour in the bakery industry. Several studies demonstrate that bread could be an excellent matrix to incorporate different functional ingredients with no major changes in the technological bakery flow [25,26,27,28,29]. Hence, an investigation was conducted on bread samples prepared from mixtures of wheat flour and FPH at replacement levels of 1.5%, 3%, 4.5%, and 6%.

## 2. Materials and Methods

### 2.1. Materials, Reagents, and Equipment Used

Fish protein hydrolysate (FPH) was delivered by SINTEF Energy, Trondheim, Norway, while the wheat flour (WF) type 650 was purchased from a local supermarket from Brașov, Romania.

The reagents used were ethyl alcohol (Eurol Industries 97, Argeș, Romania), Na_2_CO_3_ and Folin–Ciocalteu’s Reagent (VWR Chemicals, Radnor, PA, USA), Kjeldahl catalyst tablets (Merck Group, Darmstadt, Germany), H_2_SO_4_ d = 1.83–1.84 (Silver Chemicals, Bolintin Vale, Jud. Giurgiu, Romania), NaOH solution 30% and 0.1 n (Amex Lab, Bucharest, Romania), and DPPH and HCL (Sigma Aldrich–Merck Group, St. Louis, MO, USA).

The equipment used were the Metler LJ 16 thermobalance (LabMakelaar Benelux B.V., Zevenhuizen, The Netherlands), thermo-adjustable electric oven (Nabertherm GmbH, Lilienthal, Germany), Tecator Digestor Auto mineralization unit (Foss, Hilleroed, Denmark), Tecator Scrubber gas scrubbing unit (Foss, Hilleroed, Denmark), Kjeltec 2300 analysis distillation system (Tecator, Hoganas, Sweden), FOSS digestion tubes, Soxhlet extraction setup (VELP SER158/6, Multilab, Bucharest, Romania), Fibretherm-Gerhardt (C. Gerhardt GmbH & Co., Königswinter, Germany), and Mixolab Chopin+ (Chopin Technologies, Garenne, France).

### 2.2. Preparation of Composite Flours

The principal ingredient, a yellowish-white powder, was obtained by lyophilisation of the hydrolysate of the remains of cod (*Gadus morhua*). Four samples were obtained from FPH and WF mixtures as follows: P1 (98.5% wheat flour + 1.5% fish protein hydrolysate), P2 (97% wheat flour + 3% fish protein hydrolysate), P3 (95.5% wheat flour + 4.5% fish protein hydrolysate), and P4 (94% wheat flour + 6% fish protein hydrolysate), which were compared to a control sample M (100% WF).

### 2.3. Bread Making

The bread products were prepared following the procedures detailed by Oprea et al. [30]. All ingredients, WF type 650, salt, and yeast, were procured from a reputable local supermarket in Brasov, Romania. Four bread samples were obtained, using the same coding used for the flour mixtures (P1—1.5% FPH + 98.5% WF, P2—3% FPH + 97% WF, P3—4.5% FPH + 95.5% WF, and P4—6% FPH + 94% WF), which were compared to a control sample of bread obtained from 100% WF type 650. The investigated bread recipes are shown in Table 1.

The yeast suspension was prepared by combining yeast with 5 g of flour and warm water (30 °C) along with salt, dissolved in warm water (30 °C). This yeast flour mixture and salt solution were then incorporated into the flour blend, after which the combined ingredients underwent a thorough mixing process in a Silver 50 mixer (Sigma, Torbole Casaglia, Italy) for 2 min at 60 rpm, followed by an additional 8 min of 90 rpm. The initial fermentation takes place at 30 °C with a relative humidity of 70% in a specialised leavener (Telbo, NovaPan, Brașov, Romania), for a duration of 60 min. Subsequently, the dough is shaped, and a second fermentation ensues for another 35 min. The breads are than baked in a ring-type steam oven model MSR 4, (NovaPan, Romania) for 35 min at a temperature of 220 °C. Following baking, a minimum cooling period of 4 h is observed before the cutting process begins.

### 2.4. Proximate Analysis

The composition was estimated through the utilisation of the following methods: The moisture content (%) of WF and FPH flour were determined gravimetrically in triplicate, following the AACC method 44-15.02 [31]. The ash content (%) was analysed using the SR ISO Method 2171:2009 [32]. The protein content (%) was assessed according to the SR ISO Method 20483:2007 [33]. The total fat content (%) was determined using the SR Method 90:2007 [34]. The crude fibre content (%) was measured in accordance with the method outlined by Apostol et al. [35]. The sodium chloride content was determined using Mohr’s titration method [36]. The acidity of the dough was determined in accordance with SR 90/2007—Wheat flour—Methods of analysis, Method of suspension in water [34]. And finally, the carbohydrate content (g/100 g) and nutritional value (kcal/100 g) were determined based on the methodologies outlined in [29,37].

### 2.5. Physical—Chemical Properties

To assess the quality of the obtained bread samples, various characteristics were examined, including specific volume, crumb porosity, crumb elasticity, moisture, and acidity. All analytical methods employed were conducted in accordance with the guidelines outlined in STAS 91/83 [38]. Bread volume was quantified using a FORNET device, primarily assessing the volume of rapeseed displaced by the analysed product, and the results were reported as a percentage. Elasticity was determined by compressing a square piece of crumb, measuring 50 mm, into a cylindrical form for 1 min and recording its ability to return to its initial shape after compressing. The crumb porosity measurement is determined according to SR 90:2007 [34].

### 2.6. Determination of Total Phenolic Content (TPC)

The total phenolic content of the WF and FPH flour was assessed using the Folin–Ciocâlteau method as described by Obistioiu et al. [39]. The obtained results were reported as mg GAE/100 g sample and were determined in triplicate.

### 2.7. Antioxidant Activity

The antioxidant activity of the FPH flour and wheat flour was determined in triplicate by the DPPH method (2,2-diphenyl-1-picrylhydrazyl), following the protocol outlined by Ciulca et al. [40].

### 2.8. Rheological Properties

To facilitate a more accurate analysis of the impact of temperature on the dough, the rheological behaviour of the dough was studied using a state-of-the-art instrument for measuring dough properties during kneading and heat treatment. This instrument, manufactured by Chopin Technologies-France, is known as the Mixolab device. The rheological behaviour of the dough obtained from studied flour samples was analysed using the “Chopin + Protocol”, specifically following the protocol ICC No. 173 [41] for a complete characterisation of the rheological behaviour of flour. The parameters considered included dough formation time, water absorption (WA), dough stability (ST), highest torque value during mixing (C1), protein chain weakening (C2), the rate of starch gelatinisation (C3), lowest torque value (C4), torque value after cooling, baking stability (C4/C3), protein chain weakening under heating effect (α), starch gelatinisation speed (β), enzyme degradation speed (γ), and starch retrogradation during cooling (C5/C4) [42].

### 2.9. Sensory Analysis

Sensorial testing encompassed a five-point-based hedonic test and a total acceptability hedonic scale method. A panel of 20 evaluators, aged between 21 and 55 years (comprising 15 females and 5 males), was selected based on two main criteria, non-smokers and individuals in good health conditions [43,44]. The panels were selected and trained in accordance to the ISO 8586:2023 guidelines [45]. For comparison, the bread samples (P1–P4) were compared to the control sample (M). Each bread variant was cut into 2 cm thick slices and served randomly in normal temperature and light conditions.

### 2.10. Microbiological Analysis for Shelf Life

To assess shelf life, the investigated bread samples underwent microbiological analyses in accordance with the Order 27/2011 of ANSVSA for matrix of bakery products on a period of three days [44].

### 2.11. Texture Analysis

Textural analysis was determined following the methodology outlined in our previous work [30]. The textural analysis was carried out by compressing the products with the help of a piston with a diameter of 12 mm, at room temperature. Each sample was analysed three times, for three days every 24 h. The equipment was assisted by a software application that automatically calculated firmness, elasticity, cohesiveness, and gumminess.

### 2.12. Statistical Analysis

The obtained results were statistically analysed using the two-way analysis of variance (ANOVA), followed by Tukey’s HSD test, with a significance level of *p* < 0.05. Microsoft Excel 2010 (Microsoft, Redmond, WA, USA) and XLSTAT Add, soft version 15.5.03.3707 (Addinsoft, New York, NY, USA) were used to perform the mathematical and statistical analyses.

## 3. Results and Discussions

### 3.1. Chemical and Nutritional Analyses

Table 2 presents a summary of the results obtained from the chemical analyses performed according to the methods described in Section 2.

In terms of humidity, FPH flour exhibited a low value of 2.41%, a typical value taking into account the method of obtaining (lyophilisation), followed by packing in a vacuum-sealed polyethylene bag. Regarding the wheat flour, the humidity value was 13.1%, a normal value, specific to the hygroscopic balance with the ambient environment under normal conditions (20 °C, relative humidity 45%) [46]. The very high protein content of 81.21% in FPH flour is particularly noteworthy, compared to that of wheat flour of 11.37%. This stark difference opens up the possibility of fortifying various bakery products, by substituting wheat flour with hydrolysed fish protein. Similar results regarding the high protein content of FPH flour have been mentioned in numerous other studies [47,48,49,50]. According to the European Food Safety Authority (EFSA) [51,52], the population reference intakes (PRIs) vary for different demographic groups: adults require 0.83 g/kg of body weight/day, while infants/children and adolescents range from 0.83 to 1.31 g/kg of body weight/day, depending on the age. Pregnant women necessitate an additional intake of 1 g, 9 g, and 28 g per day during the first, second, and third trimesters, respectively. Breast-feeding women require an additional intake of 19 g per day during the first 6 months of lactation and 13 g per day thereafter [51]. Taking into account the water footprint of the fish protein [53,54,55], the scientific approach of recovering and valorising proteins from the waste of the fish processing industry become justifiable. The fat content of the analysed FPH flour sample was measured at 0.6%, representing approximately 50% of the value of 1.17 recorded for wheat flour, an element explained by the technology for obtaining FPH flour described in Section 2. Similar results were obtained also by Bouhamed et al. [56]. Another interesting element that emerges from Table 2 is the high content of sodium chloride. The value obtained in our case was 10.45, much higher than in the case of wheat flour (0.33), which can be explained by the nature of the raw material (sea fish) used to obtain FPH. The content of fat, crude fibre, and carbohydrates in the case of FPH exhibited significantly lower values compared to those of wheat flour. Similar results regarding the reduced fat content were also reported by Petrova et al. [57] and He et al. [58].

### 3.2. Antioxidant Activity

FPH flour and WF were studied regarding the total polyphenols and antioxidant activity (DPPH method), and the results are presented in Table 3.

Observing the TPC parameter, it becomes evident that its value in FPH flour surpassed that of WF by more than 10 times (1452.00 mg GAE/100 g vs. 126.60 mg GAE/100 g). Similarly, the antioxidant activity determined through the DPPH method was more than 50-times higher in the case of FPH flour compared to that of WF (294.00 mg Trolox/100 g vs. 5.17 mg Trolox/100 g). Similar results regarding TPC values were reported by Sharma et al. [59] and Alahmad et al. [60].

### 3.3. Determination of the Rheological Characteristics of Mixtures of WF and FPH Flour

In order to evaluate the impact of FPH flour additions on the manufacturing technologies of bakery products, an analysis was carried out using the Mixolab device. The analysis was conducted according to the ICC 173 standard [41], which measures the rheological behaviour of composite flour doughs under the influence of temperature during kneading. The testing is carried out on a properly hydrated dough in order to obtain a limit value of the consistency of the dough in the initial testing phase [42]. The results obtained when determining the rheological properties with the Mixolab device of these mixtures are presented in Table 4.

**Figure 1:**
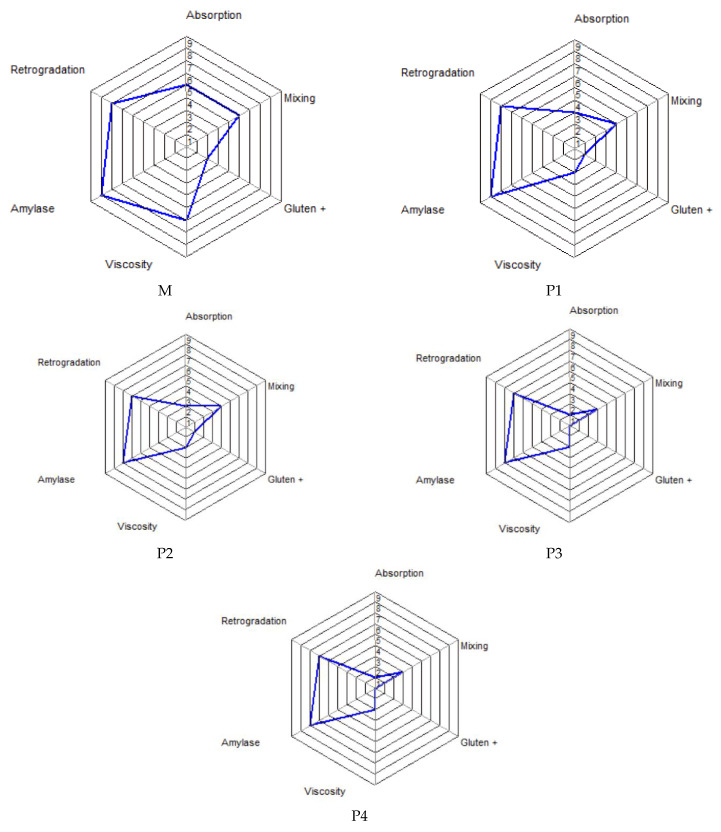
Mixolab rheological profiles of the studied flour samples (M—control sample, P1—1.5% FPH, P2—3% FPH, P3—4.5% FPH, P4—6% FPH).

As indicated in Table 4, the water absorption capacity, CH, showed a progressive decrease in all samples of wheat flour and FPH mixtures, from 58.10% (M) to a value of 52.40%. The respective mixtures are, thus, more suitable to be used for bakery products [61]. The dough stability, reflecting the dough resistance to kneading, decreased to 7.22 min in the case of the sample with 1.5% addition of FPH (P1) compared to the control sample (8.78 min). Subsequently, it progressively increased from 7.22 min (P1) to 8.33 min (P4), which indicates that the studied flour mixtures slightly surpass the optimal limit for inclusion in flours with optimal breadmaking stability [61]. The dough formation (development) time (TC1) increased from 1.20 min (M) up to 4.88 min, necessitating careful consideration in the design of the technological flow. The kneading phase of the dough in the overall bread production flow should be extended by at least 3.6 min at a low speed to allow for the correct hydration of the flours and the formation of the gluten network. The C2 parameter, gauging the relaxation of the protein chain concerning mechanical work and temperature, progressively decreases from 0.417 Nm (M) to 0.255 Nm (4A). This significant difference from the C2 value of wheat flour suggests that the incorporation of FPH significantly impacts the weakening of the wheat flour protein chain. From a technological standpoint, this element underscores the need for greater attention toward the end of mixing. Going beyond the optimal endpoint of this operation can result in significant damage to the protein chains within the dough. During the third phase, the starch gel formation occurs, at temperatures between 50 and 55 °C. At this stage, starch granules undergo volumetric expansion through water absorption, leading to an increase in dough viscosity. It is observed that the C3 parameter values decrease with the escalating replacement of wheat flour with FPH, diminishing from 1.42 to 1.27, 1.18, and 1.03 for FPH content levels of 1.5%, 3%, 4.5%, and 6%, respectively. In the case of the parameter C4, corresponding to the stability of the formed starch gel, a similar decreasing trend is noted. As the percentage of FPH increases, the C4 parameter gradually decreases, from 1.740 Nm (M) to 1.571 (P4). The stability time of the formed gel (TC4) also decreases from 30.82 min (M) to 30.16 min (P4), remaining almost constant in the other samples. This suggests that the temperature-induced softening of the dough is not significantly disrupted by the incremental increase in the percentage of FPH. The parameter C5 value indicates the degradation of the starch during the cooling phase. In our investigation, all samples from the composite bread exhibited C5 parameter values comparative to that of the control sample (M). Furthermore, these values decreased from 2.643 to 2.470 with the progressive increase in FPH content. The software application accompanying the Mixolab equipment offers, in addition to the detailed diagrams of the torque depending on temperature and time, into 6 indexes rated from 0 to 9. It profiles flour on the basis of 6 fundamental criteria, such as water absorption (WAI—water absorption index), mixing (MI—mixing index), gluten (GI—gluten index), dough maximum viscosity during warming (VI—viscosity index), starch stability (AI—amylolysis index), and starch retrogradation (RI—retrogradation index) [42]. Figure 1 shows the diagrams depicting these indicators for all analysed samples (M, P1–P4).

Table 5 presents the numerical values extracted from Figure 1 for all samples and indicators of the Mixolab profile (WAI, MI, GI, VI, AI, and RI). The WAI index exhibited a value of 5 for the control sample (M), a value of 3 for the P1 sample, a value of 2 for P2, and a value of 1 for the P3 and P4 samples. According to the Mixolab manual [42], elevated values for WAI indicate a higher absorption capacity. Therefore, the addition of FPH had a detrimental impact on dough hydration. The MI index provides information on the mechanical characteristics of the dough throughout the stages of formation, stability, and destruction of the protein network during kneading, at a temperature of 30 °C [42]. According to the Mixolab manual [42], a higher MI value correlates with increased dough stability during kneading. Therefore, the addition of FPH into the dough recipe has a marginal impact on the mechanical stability. The MI values decrease from 5 in the case of the control sample (M) to the value 4 (P1 and P2) and further to the value 3 (P3 and P4). 

The GI indicator refers to the behaviour of the gluten network during dough heating, with higher values indicating greater resistance to dough heating. In this case, Table 5 illustrates a decrease in GI values from 2 (M) to 1 (P1, P2) and then to 0 for samples P3 and P4. Overall, taking into account the MI and GI indicators, the addition of FPH reduces the characteristics of the gluten network under the combined influence of mechanical kneading and heating efforts.

VI represents an indicator of dough viscosity during the heating period, with higher values denoting increased viscosity [42]. In our study, the value of VI decreases from 6 for the control sample (M) to 2 for all samples with the addition of FPH. 

AI and RI serve as indicators of starch resistance to the amylolysis reaction and hydrolysis during Mixolab testing, respectively, with higher values indicating a reduced shelf life of the final product. In our study, it is observed that both AI and RI decrease slightly with the addition of FPH in the recipe, indicating a modest enhancement in the shelf life of the product.

### 3.4. Baking Tests to Obtain the Bread with FPH: Bread Quality

During the experimental research, five types of bread were produced using the direct method, while the coding for the experimental bread samples mirrored that of the flour mixtures, denoted as M (control sample), P1 (1.5% FPH + 98.5% WF), P2 (3% FPH + 97% WF), P3 (95.5% FPH + 4.5% WF), and P4 (6% FPH + 94% WF). The creation of these new bakery products involved employing the previously described raw materials and the same specific ingredients used in bread production for the products obtained from WF with the addition of FPH flour. Consistent with the other experiments, the same technological processes and recipes were applied. Three samples of bread were obtained from each variant, and the physicochemical indicators of these experimental bread samples produced from wheat flour with varying percentages of FPH are presented in Table 6.

The physicochemical indicators of the experimental bread samples obtained from wheat flour with different percentages of FPH are presented in Table 6. The moisture content in these bread samples obtained from WF with the addition of FPH decreases with an increase in FPH content, ranging from 43.00 (M) to 40.80 (P4). The reduction is attributed to the distinct values in the degree of WA during dough formation time and water losses during baking. The final volume of the bread depends on the expansion of the dough during baking and the ability of the matrix to stabilise the retained gases. The loaf volumes exhibit a slight insignificant decrease from 381 cm^3^ (P1) to 360 cm^3^ (P4), staying within the limits stipulated by the SR 878/96 [62] standard for white bread (minimum 280 cm^3^). The porosity of the samples aligns with the normal limits for white bread (minimum 74%), as provided in SR 878/96 [62]. The elasticity of the samples obtained from the flour mixtures does not deviate significantly from that of the control sample M. Regarding the sample, this conforms to the quality check for white bread (maximum 3.5 grd. acidity), as per the same standard with a slight increase observed in the acidity of the last two bread samples (P3 and P4). Overall, the evaluation of the physicochemical characteristics of the bread samples (Table 6) indicates that the FPH bread samples exhibit no significant change in parameters compared to those of the control sample M.

**Figure 2:**
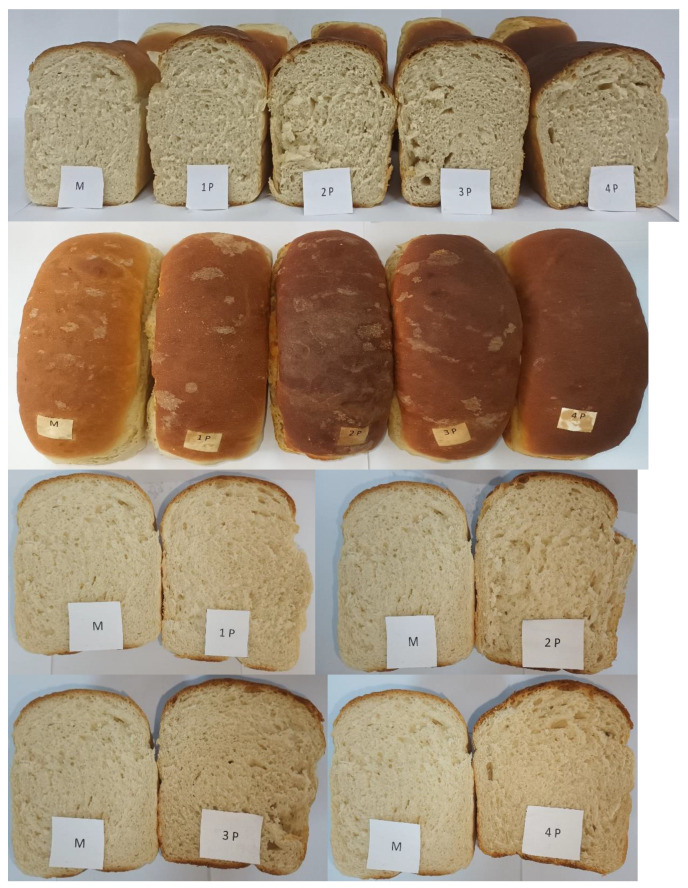
Experimental bread samples with addition of FPH—fish protein hydrolysate; M—control sample; P1—1.5% FPH=1P; P2—3% FPH=2P; P3—4.5% FPH=3P; P4—6% FPH=4P).

Figure 2 shows the experimental bread samples derived from mixtures of WF and FPH, providing a visual comparison with bread solely obtained from WF.

In contrast to the experimental bread samples incorporating other functional ingredients [63,64,65], the addition of FPH does not exert a significant influence on the colour, and both the volume and the porosity remain visibly unaffected by the addition of FPH.

### 3.5. Sensory Evaluation

To assess the acceptability of the bread samples enriched with FPH, a sensory evaluation was conducted with a panel of 20 evaluators, using a five-point scale. The average point values obtained for the bread samples with hydrolysed fish protein during the sensory evaluation are shown in Table 7.

Analysing the results of the sensory evaluation for the samples derived from blends of wheat flour and FPH (Tabel 7), it becomes evident that attributes such as crust colour, crumb colour, crumb pore uniformity, crumb softness to the touch, crumb crumbliness, salty taste, and sour taste exhibited scores closely aligned with those of the control sample. However, attributes like specific flavour and flavour persistence after chewing and swallowing registered an unfavourable increase as the percentage of FPH rose.

Utilising the total acceptability method, the following grades were obtained:

- M—obtained a score of 7.6, falling between “I like it very much” and “I like it moderately”;
- P1—obtained a score of 6.20, positioning between “I like it moderately” and “I like it slightly”;
- P2—obtained a score of 5.10, placing between “I like it easily” and “I am indifferent”;
- P3—obtained a score of 3.10, indicating “I moderately dislike”;
- P4—obtained a score of 2.00, signifying “I dislike it very much”.

From the grades presented above, it can be observed that the sample P1 exhibits acceptable attributes, while the other bread samples derived from wheat flour and FPH mixtures did not attain satisfactory scores, in comparison to the control sample. This impairment is due to the specific flavour attributes and flavour persistence after chewing and swallowing. Comparable findings were reported by Sinthusamran et al. [66].

### 3.6. Shelf Life Estimation Based on Microbiological and Water Activity

Following the guidelines of the Order 27:2011 [44], the samples underwent microbiological analysis, assessing yeasts and moulds cfu/g and monitoring water activity, over a period of 72 h. As can be seen in Table 8, all bread samples enriched with FPH fall within the stipulated regulatory limits from a microbiological point of view for at least 72 h, when stored at room temperature and packaged in a paper bag. Regarding the water activity parameter, a slight decrease is observed in all samples during storage. According to the SR ISO 21527-2:2009 [67] standard, the optimal water activity value for a bread product should be ≤0.95.

### 3.7. Texture Analysis

The assessment of the textural properties was carried out using the Instron texture analyser (model 5944, Illinois Tool Works Inc., Norwood, MA, USA), equipped with a 12 mm diameter compression piston. Cumulative results for the indicators, including firmness, cohesiveness, elasticity, and gumminess, are presented in Table 9.

Analysing the values obtained for the texture indicators, the following observations can be summarised:

- The addition of FPH led to a notable reduction in the firmness of the samples from M to P4 by approximately 40%;
- Firmness exhibits significant changes between day 1 and day 2, with a more modest increase between day 2 and day 3;
- Elasticity did not show any significant changes with the addition of FPH or over time;
- Cohesiveness remained relatively unaffected with the addition of FPH;
- Cohesiveness showed a significant decrease over time for all samples (40–50%) between day 1 and day 2, with a less pronounced decrease between day 2 and day 3;
- The gumminess of the samples decreased with FPH addition from M to P4 by approximately 40%;
- No significant changes in the sample gumminess were observed over time.

In Table 10, a schematic diagram showing the workflow, methodology, and key outcomes is provided. The left column represents the methodology carried out and used in this study; the middle column shows the workflow, starting from the raw materials to the obtained bread samples; and the right column presents the key outputs.

## 4. Conclusions

This study underscores the multifaceted impact of incorporating FPH flour into the manufacturing recipe of basic bakery products, spanning nutritional, phytochemical, rheological, organoleptic and shelf life considerations. FPH emerges as a valuable protein source capable of fortifying bakery products. Producing FPH flour by utilising by-products from the fish processing industry also represents a means to mitigate the water footprint associated with food production. It is also important to point out that the fortification of FPH flour bread is an alternative for gluten reduction in the bread, given its gluten-free composition. Phytochemical elements were also emphasised in the study, revealing elevated levels of polyphenols and an average antioxidant value. This study indicates that a high degree of substitution of wheat flour with FPH (6%) does not significantly compromise the volume and porosity. However, other organoleptic aspects such as bitter taste, aftertaste, and intense fish aroma were less appreciated by consumers. Crucially, this research identifies that a more conservative substitution such as 1.5% FPH yields bread-type bakery products rich in high-quality protein, while maintaining consumers appreciation with minimal impacts on the rheological and technological characteristics. This finding underscores the potential for achieving a harmonious balance between nutritional enhancement and consumer acceptance in the integration of FPH into bakery formulations.

## Figures and Tables

**Table 1:** Recipes for studied bread products with different substitution levels of FPH.

Samples	Ingredients				
	FPH (%)	WF Type 650 (%)	Yeast (g)	Salt (g)	Water (mL)
M	-	100	30	15	750
P1	1.5	98.5	30	15	750
P2	3	97	30	15	750
P3	4.5	95.5	30	15	750
P4	6	94	30	15	750

Note: FPH—fish protein hydrolysate; WF—wheat flour; M—control sample; P1—1.5% FPH; P2—3% FPH; P3—4.5% FPH; P4—6% FPH.

**Table 2:** Comparison between the main nutritional parameters of WF and FPH flour.

Parameter	FPH Flour	CV (%)	WF 650	CV (%)	*p*-Value (*t*-Test)
Moisture (%)	2.41 ± 0.010 ^a^	0.63	13.10 ± 0.005 ^b^	0.076	<0.0001
Ash (%)	12.73 ± 0.090 ^a^	0.74	0.66 ± 0.015 ^b^	4.009	<0.0001
Protein content (%)	81.21 ± 0.500 ^a^	0.71	11.37 ± 0.089 ^b^	1.366	<0.0001
Fat content (%)	0.60 ± 0.040 ^a^	6.66	1.17 ± 0.035 ^b^	5.199	0.0110
Carbohydrates (g/100 g)	3.03 ± 0.500 ^a^	18.69	73.70 ± 0.104 ^b^	0.245	<0.0001
Energy values (kcal/100 g)	342.00 ± 0.500 ^a^	0.16	350.80 ± 0.312 ^b^	0.089	0.0001
Raw fibre (%)	0.43 ± 0.040 ^a^	9.53	1.23 ± 0.025 ^b^	2.040	<0.0001
Sodium chloride (g/100 g)	10.45 ± 0.420 ^a^	4.02	0.33 ± 0.026 ^b^	8.010	0.0013

Note: Values marked with the same letters (a, b) do not differ significantly at *p* < 0.05 according to Tukey’s multiple test. FPH refers to fish protein hydrolysate, WF refers to wheat flour, and CV stands for the coefficient of variation.

**Table 3:** The antioxidant activity of the studied samples.

Parameter	FPH Flour	CV (%)	WF 650	CV (%)	*p*-Value (*t*-Test)
Total polyphenols (mg GAE/100 g)	1452.00 ± 36.25 ^a^	2.497	126.60 ± 2.13 ^b^	1.682	<0.0001
DPPH (mg Trolox/100 g µmol Trolox/100 g)	294.00 ± 4.79 ^a^	1.628	5.17 ± 0.05 ^b^	1.132	<0.0001

Note: Values marked with the same letters (a, b) do not differ significantly at *p* < 0.05 according to Tukey’s multiple test. FPH refers to fish protein hydrolysate, WF refers to wheat flour, and CV represents coefficient of variation.

**Table 4:** Rheological characteristics of wheat flour and FPH flour.

Parameter	M (100% WF)	P1 (98.5% WF + 1.5% FPH)	P2 (97% WF + 3% FPH)	P3 (95.5% WF + 4.5% FPH)	P4 (94% WF + 6% FPH)
Water absorption (%)	58.10 ± 0.05 ^a^	57.20 ± 0.03 ^b^	56.00 ± 0.04 ^c^	54.70 ± 0.01 ^d^	52.40 ± 0.02 ^e^
Stability (min)	8.78 ± 0.28 ^a^	7.22 ± 0.22 ^b^	7.52 ± 0.19 ^c^	7.60 ± 0.12 ^d^	8.33 ± 0.11 ^e^
Amplitude (Nm)	0.091 ± 0.01 ^a^	0.078 ± 0.01 ^b^	0.072 ± 0.01 ^c^	0.088 ± 0.01 ^d^	0.073 ± 0.01 ^c^
Moisture (%)	11.90 ± 0.5 ^a^	12.30 ± 0.02 ^b^	12.40 ± 0.03 ^c^	12.30 ± 0.01 ^b^	12.20 ± 0.04 ^d^
α (Nm/min)	−0.080 ± 0.002 ^a^	−0.070 ± 0.002 ^b^	−0.084 ± 0.003 ^a^	−0.076 ± 0.003 ^b^	−0.090 ± 0.002 ^c^
β (Nm/min)	0.112 ± 0.003 ^a^	0.278 ± 0.004 ^b^	0.234 ± 0.004 ^c^	0.152 ± 0.003 ^d^	0.106 ± 0.003 ^a^
γ (Nm/min)	−0.024 ± 0.003 ^a^	−0.062 ± 0.005 ^d^	0.036 ± 0.003 ^b^	0.050 ± 0.004 ^c^	0.064 ± 0.001 ^d^
C1	1.132 ± 0.01 ^a^	1.115 ± 0.02 ^b^	1.058 ± 0.01 ^c^	1.078 ± 0.03 ^d^	1.075 ± 0.04 ^d^
TC1	1.20 ± 0.1 ^a^	4.07 ± 0.07 ^b^	4.10 ± 0.08 ^b^	4.25 ± 0.06 ^c^	4.88 ± 0.05 ^d^
C2	0.417 ± 0.01 ^a^	0.350 ± 0.01 ^b^	0.294 ± 0.01 ^c^	0.275 ± 0.02 ^d^	0.255 ± 0.01 ^e^
TC2	17.37 ± 0.13 ^a^	17.60 ± 0.11 ^b^	18.03 ± 0.12 ^c^	17.92 ± 0.13 ^d^	18.00 ± 0.11 ^c,d^
C3	1.793 ± 0.02 ^a^	1.422 ± 0.01 ^b^	1.273 ± 0.02 ^c^	1.181 ± 0.01 ^d^	1.035 ± 0.01 ^e^
TC3	27.95 ± 0.32 ^a^	23.00 ± 0.28 ^b^	24.08 ± 0.29 ^c^	24.56 ± 0.28 ^c,d^	25.02 ± 0.28 ^d^
C4	1.740 ± 0.01 ^a^	1.672 ± 0.01 ^b^	1.568 ± 0.01 ^c^	1.584 ± 0.01 ^d^	1.571 ± 0.01 ^c,d^
TC4	30.82 ± 0.17 ^b^	30.24 ± 0.13 ^a^	30.28 ± 0.15 ^a^	30.88 ± 0.10 ^b^	30.16 ± 0.11 ^a^
C5	2.731 ± 0.08 ^a^	2.643 ± 0.09 ^b^	2.576 ± 0.10 ^c^	2.477 ± 0.11 ^d^	2.470 ± 0.12 ^d^
TC5	45.00 ± 0.01 ^a^	45.02 ± 0.01 ^a^	45.02 ± 0.01 ^a^	45.00 ± 0.01 ^a^	45.00 ± 0.01 ^a^

a, b, c, d, e—values marked with the same letters do not differ significantly at *p* < 0.05 according to Tukey’s multiple test. FPH—fish protein hydrolysate; WF—wheat flour; M—control sample; P1—1.5% FPH; P2—3% FPH; P3—4.5% FPH; P4—6% FPH; α—protein chain weakening under heating effect; β—starch gelatinisation speed; γ—enzyme degradation speed; C1—highest torque value during mixing; C2—protein chain weakening; C3—the rate of starch gelatinisation; C4—lowest torque value; C5—starch retrogradation; TCi—time corresponding to Ci (i = 1 … 5).

**Table 5:** The main indicators of the Mixolab profile of the studied samples.

Samples	WAI	MI	GI	VI	AI	RI
M	5	5	2	6	8	7
P1	3	4	1	2	8	7
P2	2	4	1	2	7	6
P3	1	3	0	2	7	6
P4	1	3	0	2	7	6

Note: WAI—water absorption index; MI—mixing index; GI—gluten index; VI—viscosity index; AI—amylolysis index; RI—retrogradation index;; M—control sample; P1—1.5% FPH; P2—3% FPH; P3—4.5% FPH; P4—6% FPH.

**Table 6:** Physicochemical indicators of experimental breads with added FPH.

Sample	Analysis					
	Mass (kg)	Moisture (%)	Acidity (Degree)	Specific Volume (cm^3^/100 g)	Porosity (%)	Elasticity (%)
M	0.453 ± 0.03 ^a^	43.00 ± 0.9 ^a^	1.8 ± 0.02 ^a^	381 ± 3.16 ^a^	83 ± 1.63 ^a^	95 ± 0.93 ^a^
P1	0.451 ± 0.02 ^a^	42.06 ± 0.8 ^b^	2.0 ± 0.01 ^b^	376 ± 3.34 ^b^	83 ± 1.62 ^a^	97 ± 0.94 ^b^
P2	0.449 ± 0.02 ^a^	41.44 ± 0.8 ^c^	2.2 ± 0.02 ^c^	373 ± 3.28 ^b^	84 ± 1.66 ^b^	95 ± 0.92 ^a^
P3	0.454 ± 0.02 ^a^	40.84 ± 0.8 ^d^	2.8 ± 0.03 ^d^	371 ± 3.33 ^b^	83 ± 1.64 ^a^	95 ± 0.93 ^a^
P4	0.451 ± 0.02 ^a^	40.80 ± 0.8 ^e^	2.8 ± 0.03 ^d^	360 ± 3.42 ^c^	81 ± 1.67 ^c^	97 ± 0.94 ^b^

Note: Values marked with the same letters (a, b, c, d, e) do not differ significantly at *p* < 0.05, in accordance with Tukey’s multiple test. The samples are M—control sample, P1—1.5% FPH, P2—3% FPH, P3—4.5% FPH, P4—6% FPH.

**Table 7:** Centralizer of the scores obtained for the bread samples with added FPH through the analysis conducted with the panel of evaluators.

Sensorial Attributes	M	P1	P2	P3	P4
Crust colour	2.80 ± 0.04 ^a^	2.80 ± 0.05 ^a^	3.00 ± 0.06 ^b^	3.05 ± 0.08 ^b^	3.40 ± 0.06 ^c^
Crumb colour	1.70 ± 0.03 ^a^	1.75 ± 0.04 ^a^	1.80 ± 0.08 ^b^	2.00 ± 0.03 ^c^	2.30 ± 0.02 ^d^
Crumb pore uniformity	3.60 ± 0.20 ^a^	3.60 ± 0.21 ^a^	2.85 ± 0.39 ^b^	2.90 ± 0.28 ^b,c^	3.00 ± 0.25 ^c^
Crumb softness	3.55 ± 0.07 ^a^	3.60 ± 0.06 ^a^	3.55 ± 0.04 ^a^	3.55 ± 0.04 ^a^	3.55 ± 0.04 ^a^
Crumb crumbliness	4.20 ± 0.07 ^a^	4.00 ± 0.08 ^b^	3.90 ± 0.06 ^c^	3.90 ± 0.06 ^c^	4.00 ± 0.08 ^b^
Bitter taste	1.80 ± 0.02 ^a^	2.60 ± 0.04 ^b^	2.70 ± 0.06 ^b,c^	2.80 ± 0.09 ^c^	3.20 ± 1.20 ^d^
Salty taste	2.05 ± 0.05 ^a^	2.65 ± 0.07 ^b^	2.65 ± 0.07 ^b^	2.65 ± 0.06 ^b^	2.65 ± 0.06 ^b^
Sour taste	1.30 ± 0.02 ^a^	1.80 ± 0.04 ^b^	1.85 ± 0.02 ^b^	1.85 ± 0.03 ^b^	1.85 ± 0.03 ^b^
Specific aroma	2.50 ± 0.05 ^a^	3.30 ± 0.07 ^b^	4.30 ± 0.09 ^c^	4.35 ± 1.10 ^d^	5.00 ± 1.60 ^e^
Persistence of flavour after chewing and swallowing	1.90 ± 0.03 ^a^	3.10 ± 0.04 ^b^	4.20 ± 0.05 ^c^	4.30 ± 0.05 ^c^	5.00 ± 0.7 ^d^

Note: Values marked with the same letters (a, b, c, d, e) do not differ significantly at *p* < 0.05 in accordance with Tukey’s multiple test.

**Table 8:** Microbiological indicators of bread samples with the addition of FPH.

Sample	Yeasts and Moulds cfu/g	Water Activity (Aw)
Initial analysis		
M	<10	0.966 ± 0.018 ^a^
P1	<10	0.966 ± 0.018 ^a^
P2	<10	0.970 ± 0.018 ^b^
P3	<10	0.967 ± 0.018 ^a^
P4	<10	0.967 ± 0.018 ^a^
Analysis after 48 h		
M	<10	0.952 ± 0.019 ^a^
P1	<10	0.962 ± 0.019 ^b^
P2	<10	0.963 ± 0.018 ^b^
P3	<10	0.949 ± 0.019 ^a^
P4	<10	0.955 ± 0.018 ^a^
Analysis after 72 h		
M	<10	0.951 ± 0.018 ^a^
P1	<10	0.936 ± 0.018 ^b^
P2	<10	0.954 ± 0.019 ^c^
P3	<10	0.947 ± 0.019 ^a^
P4	<10	0.954 ± 0.018 ^c^

Note: Values marked with the same letters (a, b, c) do not differ significantly at *p* < 0.05 in accordance with Tukey’s multiple test. FPH represents fish protein hydrolysate, and the samples are given as M—control sample, P1—1.5% FPH, P2—3% FPH, P3—4.5% FPH, P4—6% FPH.

**Table 9:** Textural analysis of bread samples with the addition of fish protein hydrolysate.

Analysis	Time	Samples				
		M	P1	P2	P3	P4
Firmness (Force 40%) (N)	Day 1	1.62 ± 0.17 ^a,A^	1.24 ± 0.08 ^a,B^	1.25 ± 0.14 ^a,B^	1.26 ± 0.06 ^a,B^	1.13 ± 0.05 ^a,C^
	Day 2	2.09 ± 0.16 ^b,A^	1.57 ± 0.15 ^b,B^	2.06 ± 0.22 ^b,A^	2.07 ± 0.02 ^b,A^	2.42 ± 0.03 ^b,C^
	Day 3	2.34 ± 0.28 ^c,A^	1.59 ± 0.31 ^c,B^	2.24 ± 0.40 ^c,C^	2.22 ± 0.07 ^c,C^	2.64 ± 0.12 ^c,D^
Cohesiveness	Day 1	0.70 ± 0.01 ^a,A^	0.73 ± 0.02 ^a,A^	0.71 ± 0.01 ^a,A^	0.68 ± 0.03 ^a,A^	0.68 ± 0.06 ^a,A^
	Day 2	0.50 ± 0.10 ^b,A^	0.59 ± 0.03 ^b,C^	0.52 ± 0.05 ^b,A^	0.53 ± 0.04 ^b,B,C^	0.46 ± 0.03 ^b,A^
	Day 3	0.47 ± 0.03 ^b,B^	0.56 ± 0.04 ^b,C^	0.49 ± 0.06 ^b,C^	0.41 ± 0.08 ^c,B^	0.26 ± 0.02 ^c,A^
Elasticity	Day 1	0.99 ± 0.01 ^a,A^	0.98 ± 0.02 ^a,A^	0.98 ± 0.01 ^a,A^	0.98 ± 0.01 ^a,A^	0.99 ± 0.01 ^a,A^
	Day 2	0.98 ± 0.02 ^a,A^	1.00 ± 0.02 ^a,A^	0.98 ± 0.01 ^a,A^	0.97 ± 0.02 ^a,A^	0.99 ± 0.01 ^a,A^
	Day 3	0.98 ± 0.01 ^a,A^	0.98 ± 0.01 ^a,A^	0.98 ± 0.02 ^a,A^	0.97 ± 0.02 ^a,A^	0.99 ± 0.01 ^a,A^
Gumminess (N)	Day 1	1.12 ± 0.14 ^a,C^	0.89 ± 0.08 ^a,B^	0.88 ± 0.08 ^a,B^	0.84 ± 0.09 ^a,B^	0.76 ± 0.09 ^a,A^
	Day 2	1.02 ± 0.12 ^b,B^	0.92 ± 0.13 ^a,A^	0.94 ± 0.08 ^b,A^	0.96 ± 0.12 ^b,B^	0.79 ± 0.07 ^a,A^
	Day 3	1.07 ± 0.18 ^b,D^	0.87 ± 0.11 ^a,B^	0.98 ± 0.18 ^b,C^	0.88 ± 0.13 ^a,B^	0.67 ± 0.14 ^b,A^

Note: Values marked with the same letters (a, b, c) in a column on a period of three days do not differ significantly at *p* < 0.05 according to Tukey’s multiple test. Values marked with the same letters (A, B, C, D) in a row do not differ significantly at *p* < 0.05 according to Tukey’s multiple test. The samples are given as M—control sample, P1—1.5% FPH, P2—3% FPH, P3—4.5% FPH, P4—6% FPH.

**Table 10:** Schematic diagram of the workflow and methodology used in the study.

Methodology Chemical, nutritional, and antioxidant analyses of the studied raw materials	Workflow 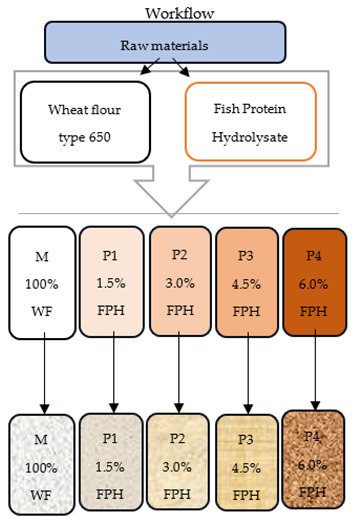	Key outputs: Chemical and nutritional characteristics: moisture, ash, protein content, fat content, carbohydrates, energy values, raw fibre, sodium chloride; Antioxidant activity: total polyphenols; DPPH activity.
Rheological and enzymatic analyses of the studied flour mixtures with different substitution degrees		Outputs: Rheological and enzymatic characteristics: water absorption, stability, amplitude, moisture, α, β, γ, C1, TC1, C2, TC2, C3, TC3, C4, TC4, C5, TC5, rheological profile (WAI, MI, GI, VI, AI, RI).
Physicochemical, sensorial microbiological, and textural analyses of the bread samples		Outputs: Physicochemical characteristics: mass, moisture, acidity, specific volume, porosity, elasticity; Sensorial characteristics: crust and crumb colour, crumb pore uniformity, crumb softness, crumb crumbliness, bitter, salty, sour taste, specific aroma, persistence of flavour after chewing and swallowing; Microbiological: yeasts and moulds, water activity; Textural characteristics: firmness, cohesiveness, elasticity, gumminess.

## Data Availability

The original contributions presented in this study are included in the article; further inquiries can be directed to the corresponding author.
